# Metabolic anomalies in vitiligo: a new frontier for drug repurposing strategies

**DOI:** 10.3389/fphar.2025.1546836

**Published:** 2025-04-15

**Authors:** Alessia Paganelli, Federica Papaccio, Mauro Picardo, Barbara Bellei

**Affiliations:** ^1^ IDI-IRCCS Istituto Dermopatico dell’Immacolata, Rome, Italy; ^2^ Cutaneous Physiopathology and Integrated Center of Metabolomics Research, San Gallicano Dermatological Institute, IRCCS, Rome, Italy

**Keywords:** vitiligo, metabolic syndrome, mitochondrial damage, oxidative stress, lipid metabolism, glucose, insulin, PPAR γ

## Abstract

Vitiligo is a chronic autoimmune condition characterized by the destruction of melanocytes, leading to patchy loss of skin depigmentation. Although its precise cause remains unclear, recent evidence suggests that metabolic disturbances, particularly oxidative stress and mitochondrial dysfunction, may play a significant role in the pathogenesis of the disease. Oxidative stress is thought to damage melanocytes and trigger inflammatory responses, culminating in melanocyte immune-mediate destruction. Additionally, patients with vitiligo often exhibit extra-cutaneous metabolic abnormalities such as abnormal glucose metabolism, dyslipidemia, high fasting plasma glucose levels, high blood pressure, out of range C-peptide and low biological antioxidant capacity, suggesting a potential link between metabolic impairment and vitiligo development. This implies that the loss of functional melanocytes mirrors a more general systemic targetable dysfunction. Notably, therapies targeting metabolic pathways, particularly those involving mitochondrial metabolism, such as the peroxisome proliferator-activated nuclear receptor γ (PPARγ) agonists, are currently being investigated as potential treatments for vitiligo. PPARγ activation restores mitochondrial membrane potential, mitochondrial DNA copy number and, consequently, ATP production. Moreover, PPARγ agonists counteract oxidative stress, reduce inflammation, inhibit apoptosis, and maintain fatty acid metabolism, in addition to the well-known capability to enhance insulin sensitivity. Additionally, increasing evidence of a strong relationship between metabolic alterations and vitiligo pathogenesis suggests a role for other approved anti-diabetic treatments, like metformin and fibrates, in vitiligo treatment. Taken together, these data support the use of approaches alternative to traditional immune-suppressive treatments for the treatment of vitiligo.

## 1 Introduction

Vitiligo is an acquired immune-mediated chronic skin condition characterized by skin depigmentation secondary to melanocyte depletion. In its most frequent form, non-segmental vitiligo, skin lesions are represented by milky-white depigmented macules with sharply demarcated borders and variable dimensions and distribution (Alikhan et al., 2011).

The prevalence of vitiligo is quite variable worldwide, with recent estimates indicating a global prevalence of approximately 0.5%–2% (Zhang et al., 2016; Harris et al., 2020). However, regional variations have been documented so far. All age groups can potentially be affected, with mean age at diagnosis ranging from 20 to 30. Despite its asymptomatic and non-life-threatening nature, vitiligo deeply impacts on patients’ quality of life due to its heavy psychological burden, especially when dealing with women, adolescents, and psychiatric patients (Picardo et al., 2015; Fekete et al., 2024). Disease localization, progression, and response to treatment are highly variable (Seneschal et al., 2021) probably because of the incomplete characterization of pathogenic mechanisms.

The etiology of the disease is multifactorial, with both genetic and environmental factors playing a role in triggering the autoimmune response against melanocytes. Recently, significant advancements have been made in understanding vitiligo pathophysiology, with extensive characterization of the immunological bases (Seneschal et al., 2021; Martins et al., 2022). As for the immunological mechanisms, interferon-γ (IFNγ) secreting CD8^+^ cytotoxic T cells are renowned key actors in melanocyte destruction. However, a role in innate immunity has also been documented. Dendritic cells, macrophages, and NK (natural killer) cells have been described in the skin of vitiligo patients (Yu et al., 2012; Tulic et al., 2019; Gholijani et al., 2020). Recent literature suggests that the activation of innate immunity is possibly triggered by damaged melanocytes secondary to oxidative stress: such pro-inflammatory milieu promotes cytokine secretion and antigen presentation, resulting in adaptive immune system activation, with autoreactive T-cells amplifying damage to melanocytes (Speeckaert et al., 2024). Vitiligo affects skin melanocytes, but additional markers of the disease have been documented in keratinocytes, dermal fibroblasts and extra-cutaneous cells, implying a systemic influence (Wu et al., 2024; Dell’Anna et al., 2010; Kovacs et al., 2022). Moreover, peripheral blood mononuclear cells (PBMCs) display flags of oxidative imbalance and defective lipid arrangement (Dell’Anna et al., 2010) as well as a peculiar miRNA expression pattern (Wang et al., 2015).

The present paper aims at providing an overview of the metabolic changes, both at a cellular and systemic level, linked to melanocyte damage and therefore to the auto-immune activation in vitiligo.

**FIGURE 1:**
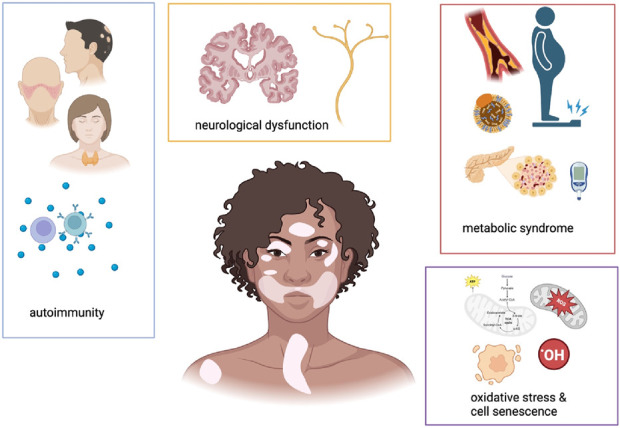
Schematic representations of main pathological alterations associated with vitiligo. This cartoon underscores that patients affected by vitiligo often suffer from autoimmune, neurological and metabolic comorbidities. A common pathogenetic mechanism at a cellular level resides in cell senescence and mitochondrial dysfunction, leading to altered cell metabolic status. Created with https://www.BioRender.com.

An overview in the main altered mechanisms associated with vitiligo is provided in Figure 1.

## 2 Comorbidities in vitiligo patients, general considerations

Epidemiological data reveal a significant number of comorbidities associated with vitiligo, although prevalence rates vary according to race, area, sex, and age of the studied population. These include a wide range of disorders, either of dermatological interest or not. More recently, several studies have proven a link between vitiligo and metabolic disturbances, suggesting that both immunological and non-immunological components are involved in the pathogenesis of vitiligo. The assessment of comorbidities in vitiligo paves the way to the definition of innovative therapeutic approaches.

### 2.1 Autoimmune comorbidities

Patients with vitiligo are more likely to have cutaneous and extra-cutaneous immune-mediated comorbidities compared to the general population. A cross-sectional study in 2023 reported that 17.7% of the patients with vitiligo had one or more autoimmune comorbidities (Rios-Duarte et al., 2023). Globally, the most frequently reported comorbid disorders were thyroid disease, including Hashimoto thyroiditis and Grave’s disease, alopecia areata, atopic dermatitis, psoriasis, systemic lupus erythematosus (SLE) and rheumatoid arthritis. In another study including 11,412 patients, the more commonly associated disorder was atopic dermatitis, followed by Hashimoto’s thyroiditis, whereas alopecia areata was the second most frequent dermatological comorbidity (Ramot et al., 2024). Of note, depending on the population studied, minor frequency differences among comorbidities have been reported (Laberge et al., 2005; Ayanlowo et al., 2009; Zhang et al., 2009). With regard to cutaneous comorbid disorders, the largest effect sizes have been demonstrated for alopecia areata and SSc. However, also psoriasis, atopic dermatitis, lichen sclerosus (LS) and linear morphea also co-occur frequently with vitiligo (Kridin et al., 2023; Yen and Chi, 2019; Mohan and Silverberg, 2015). It is important to consider that white patches in LS may occasionally be confused with vitiligo, although the concomitant complete loss of melanocytes may develop in some cases of advanced LS. To differentiate between the two conditions, an immunohistochemical study indicated melanocyte count as a possible discriminant, since melanocytes are completely absent in vitiligo but only reduced in LS (Park et al., 2023). Reflectance confocal microscopy has also been proposed for such aim and demonstrated good results in distinguishing LS from vitiligo (Gu et al., 2022).

Most comorbidities are diagnosed within 1–3 years from vitiligo diagnosis. On the contrary, alopecia areata and vitiligo mostly appear simultaneously, indicating that the two diseases could share the exact pathogenetic mechanism (Ezzedine et al., 2023). In a set of US cases, the most frequently observed autoimmune comorbidities in patients with vitiligo were type 1 diabetes (4.5%), rheumatoid arthritis (2.8%), SLE (2.5%), autoimmune thyroiditis (1.6%), Addison’s disease (1.1%), systemic sclerosis (SSc; 0.9%), pernicious anemia (0.7%), myasthenia gravis (0.7%), Sjögren’s syndrome (0.6%), and Crohn’s disease (0.6%) (Rios-Duarte et al., 2023). The common trait among these different tissue-specific autoimmune disorders is likely the Th1-prevalent autoimmune response associated with elevated CXCL10 circulating level and reduced number of Tregs underlying a possible common pathogenic mechanism (Hu and Wang, 2023).

Genome-wide association studies (GWAS) underscored a clear genetic background shared between vitiligo and other autoimmune diseases (Márquez and Martín, 2022). In particular, the identification of genes encoding for proteins involved in antigen processing and presentation, as well as in immune cell activation, suggests a common autoimmune triggering mechanism (Spritz and Santorico, 2021). However, other factors could also play a role in this shared background. Bioinformatic analysis of genes involved in both psoriasis and vitiligo, for example, identified 164 common genes. Interestingly, these genes are involved in the dynamic involvement of microtubules and microfilaments, organelle trafficking and immune checkpoint activity (Wang et al., 2024).

### 2.2 Oncological aspects of vitiligo comorbidities

Epidemiological data indicate that vitiligo patients have a diminished overall incidence rate of cancer (Bae et al., 2019; Weng et al., 2021), suggesting that a general, nonspecific activated state of immune surveillance seen in autoimmunity may confer protection against cancer.

Despite the impaired protection from UV rays due to lack of melanin, patients with vitiligo display lower incidence of non-melanoma skin cancers (squamous and basal cell carcinomas) (Schallreuter et al., 2008; Ramot et al., 2024).

Moreover, significant resistance to UV damage is appreciable at a molecular level, as indicated by the presence of a low UV-regulated transcriptome and an anti-tumoral molecular signature (Singh et al., 2021). Both immunological and non-immunological pathways (particularly p53 signaling) might explain the lower risk of skin cancer in vitiligo subjects (Rooker et al., 2024). Similarly, a decreased incidence of melanoma has also been documented by some authors (Paradisi et al., 2014; Ferguson et al., 2023). However, conflicting data have been published so far in this setting (Han et al., 2023). Moreover, the relationship between vitiligo and melanoma is complicated by the genetic background: specific alleles in genes related to T cell responses directed against melanocyte or melanoma-specific antigens can enhance host anti-tumor immune responses when immunotherapy is administered (Guida et al., 2021).

### 2.3 Neurological comorbidities

Given that melanocytes are not only present in the skin but can also be found in the iris (eye), cochlea, mesencephalon, leptomeninges, heart, and adipose tissue (Ikeda et al., 2021; Page et al., 2011), vitiligo comorbidities may be a direct consequence of melanocyte damage in different tissues and organs (Plonka et al., 2009). Indeed, skin melanin levels seem to directly correlate with total body melanin. This trend has been confirmed by recent studies indicating that darker skin phototypes are associated with lower prevalence of hearing loss. Melanin is crucial for ROS scavenging, metal absorption, thermal regulation, drug uptake, immune functions, and energy transduction.

Neuromelanin is a neuronal pigment composed of both eumelanin and pheomelanin that can be found mainly in brain regions enriched in dopaminergic or noradrenergic neurons, brain components highly exposed to oxidative stress. Neuromelanin function is mostly considered protective against oxygen-reactive species toxicity. Due to the common embryologic origin (neural crest), common pathological mechanisms and ease of access, skin melanocytes have been proposed as a model for the study of neurodegenerative diseases.

Recently, several studies have assessed the association between vitiligo and the risk of neurodegenerative diseases Chang et al., 2019; Kridin et al., 2024; Chang et al., 2022; Wu et al., 2024). This association could be explained by chronic oxidative stress (Naspi et al., 2014), the pro-inflammatory environment not restricted to the skin and the loss of neuromelanin.

On the other hand, other authors investigated the presence of abnormalities (such as axonal degeneration) of dermal nerves in vitiligo skin.

Beyond strictly neurological alterations, patients with vitiligo may experience an increased risk for mental health conditions, including depression and anxiety. This is partly due to the visible nature of vitiligo, which can lead to significant psychosocial distress, low self-esteem, social isolation and even sleep disturbances.

Mental disorders are not necessarily secondary to cosmetic issues. In fact, psychiatric biomarkers such as brain-derived neurotrophic factor (BDNF) and corticotropin-releasing hormone (CRH) have been found to be down and upregulated respectively in vitiligo patients. Psychological stress itself is a major factor in inducing and exacerbating vitiligo creating a loop that sometimes affects treatment efficacy.

## 3 Metabolic comorbidities

Diabetes is a well-known comorbidity in vitiligo (Chang et al., 2019; Hu and Wang, 2023. Precisely, vitiligo seems to be causally related to type 1 diabetes (Hu and Wang, 2023). Phenotypically, the immune-mediated destruction of functional pancreatic β-cell of type 1 diabetes resembles the selective destruction of skin melanocytes in vitiligo patients.

However, epidemiological and genetic proof of a significant correlation between diabetes and vitiligo is not restricted to type 1 but also involves type 2 diabetes (Chang et al., 2019). Shared genetic alterations in genes encoding for proteins involved in pancreatic secretion, such as ADCY5, CTRB1, CTRB2, and KCNQ1, likely confirm a role of glucose impairment in vitiligo pathogenesis.

There is growing evidence that individuals affected by vitiligo may have an increased prevalence of metabolic syndrome, which is a cluster of conditions including insulin resistance, dyslipidemia, obesity, and hypertension (Ataş and Gönül, 2017; Chang et al., 2019; Tanacan and Atakan, 2020; Tanacan and Atakan, 2020). In fact, vitiligo patients also often show abnormal lipid profiles, including elevated levels of low-density lipoprotein (LDL) and reduced high-density lipoprotein (HDL) (Mustafa et al., 2023; Papaccio et al., 2024). A positive correlation between vitiligo disease activity (VIDA) score and serum cholesterol level, LDL and LDL/HDL ratio has recently been demonstrated (Shaker et al., 2022). Not surprisingly, desnutrin plasma levels are decreased in vitiligo subjects compared to controls, in line with the central role of such lipase in adipose tissue lipolysis. These data support the impaired fasting glucose and enhanced insulin secretion previously demonstrated in vitiligo patients (Karadag et al., 2011). Notably, dyslipidemia and traditional risk factors like diabetes, hypertension, or smoking activate the NADPH oxidase system, leading to excessive superoxide anion production and promoting oxidative stress. Of note, a correlation between metabolic abnormalities and disease activity and extension has been documented (Bathina et al., 2024). Consistently, previous studies reported that lipid-lowering drugs can reverse the depigmentation of vitiligo (Agarwal et al., 2015; Noël et al., 2004).

Some studies suggested altered levels of homocysteine and cysteine in vitiligo patients (Liang et al., 2024; Tsai et al., 2019). When elevated, homocysteine induces oxidative stress and endothelial dysfunction, at the same time inhibiting tyrosinase activity. (Ataş et al., 2015).

Local alterations of metabolism also seem to be responsible for dendritic cell activation in halo nevi (Jiang et al., 2024). The inflammatory infiltrate leading to melanocyte destruction is mostly composed of T cells. Not surprisingly, halo nevi are associated with the occurrence of autoimmune diseases, including vitiligo (van Geel et al., 2012; Patrizi et al., 2013).

## 4 Cellular and molecular mechanisms of metabolic disturbances in vitiligo

Abnormal glucose metabolism in vitiligo results from an imbalanced oxidative stress response typical of low-grade inflammation associated with chronic disorders. A possible explanation for this phenomenon resides in the low levels of antioxidants, including catalase (CAT), glutathione peroxidase (GPx), glucose-6-phosphate dehydrogenase (G6PD) and superoxide dismutase (SOD), in skin lesions and serum of patients with vitiligo (Yildirim et al., 2003; Hazneci et al., 2005; Shajil and Begum, 2006; Laddha et al., 2013; Agrawal et al., 2015). Defective mitochondrial OXPHOS complexes determine higher ROS generation, low ATP production and collapsed mitochondrial membrane potential. Concerning cellular energetic metabolism, several studies highlighted impaired intracellular ATP production in vitiligo melanocytes (Dell’Anna et al., 2017). However, besides being an intracellular energy source, ATP also functions as an alarmin and promotes inflammation when released from cells. Treatment of melanocytes with high ATP concentrations has been demonstrated to result in cell death, which could imply a role for ATP in melanocyte death in vitiligo (Dosch et al., 2018; Lee E. J. et al., 2019; Ahn et al., 2020). Impaired mitochondrial function has also been associated with several vitiligo comorbidities such as (AD), multiple sclerosis (Sadeghian et al., 2016) and SLE, pointing at metabolic dysregulation as a shared pathogenic mechanism. Stabilization of mitochondrial lipid components, particularly cardiolipin, by MTP-131 supplementation, has been showed to restore mitochondrial function *in vitro*, suggesting new potential therapeutic approaches for vitiligo (Chen et al., 2017).

Metabolic impairment in the setting of vitiligo, however, is not limited to glucose but also involves lipid metabolism (Karadag et al., 2011; Chuang and Chang, 2022; Kang et al., 2022). Lipids play a crucial role in cellular membranes and are central to oxidative stress processes. *In vitro*, vitiligo melanocytes have higher amounts of oxysterols, in particular 7-beta-hydroxycholesterol and 7-ketocholesterol (Bellei et al., 2013). These cholesterol oxidation products have important physiological roles, including cholesterol homeostasis and oxysterols-induced cell death that can take part in degenerative pathologies (Gamba et al., 2012). Relatively recent metabolomic studies revealed *de novo* fatty acid synthesis and mitochondrial tricarboxylic acid cycle to have a deep impact on vitiligo pathogenesis (Sahoo et al., 2017). Pietrzak et al. also proposed a role for lipid peroxidation in contributing to oxidative damage (Pietrzak et al., 2012). Our group recently demonstrated that polyunsaturated fatty acids (PUFA) composition is altered in vitiligo patient.

Senescent melanocytes exhibit a specific “senescence-associated secretory phenotype” (SASP) possibly attracting immune cells responsible for melanocyte clearance (Bellei et al., 2013; Ohtani, 2019; Lee et al., 2021). However, recent evidence underscores how cellular damage is not limited to melanocytes in vitiligo. Impaired redox balance and altered senescence marker expression have also been described in cultured lesional keratinocytes, as well as in non-lesional epidermis from vitiligo patients (Bondanza et al., 2007; Kostyuk et al., 2010; Bellei et al., 2013). Keratinocytes from subjects affected by vitiligo, even in non-lesional areas, undergo an impaired differentiation process compared to healthy individuals, with an altered composition of epidermal lipids in terms of ceramides-to-free fatty acid ratio (Kovacs et al., 2022). Spontaneous release of pro-inflammatory cytokines and chemokines has also been observed in vitiligo keratinocytes.

**FIGURE 2:**
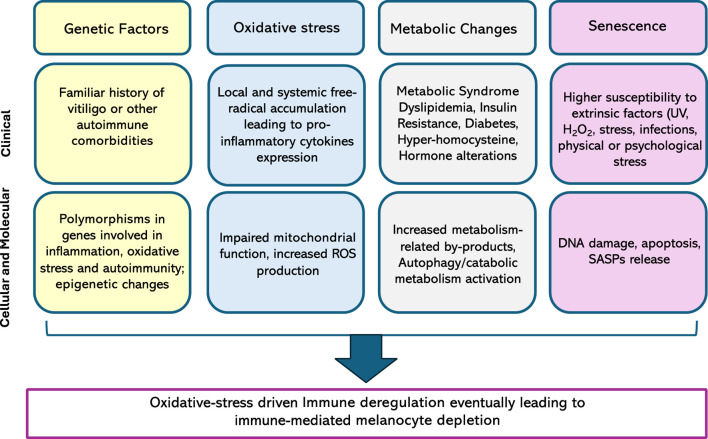
Main alterations contributing to vitiligo pathogenesis, both at a clinical and a biological level. ROS, reactive oxygen species, UV, ultraviolet; SASP, senescence-associated secretory phenotype.

Nonetheless, fibroblasts also seem to be altered in vitiligo patients, both in lesional and non-lesional skin. In fact, higher increased basal ROS levels, upregulation of the stress-induced marker p53, and a myofibroblast-like phenotype are prominent features of the disease. Fibroblast-related SAPS seems to alter the expression of E-cadherin on keratinocytes, which ultimately leads to the detachment of melanocytes following minor stress stimuli (Kovacs et al., 2018). Main pathogenetic mecahnisms in vitiligo are summarized in Figure 2.

## 5 Serum markers of metabolic impairment in vitiligo patients: experience from a tertiary referral center

Retrospective evaluation of clinical health records of vitiligo patients referred to our center between January and June 2023 was performed. Collected data included: demographic and clinical information (disease duration, stability, familiarity, extension, therapy), comorbidities, available basal blood test results (folic acid, vitamin D, ESR, fasting and post-prandial glucose and insulin, HDL and LDL cholesterol, triglycerides). Blood tests performed Disease stability was defined according to the following criteria: no new lesions in the last 12 months, no Koebner sign, no trichrome or confetti-like lesions. In the presence of one or more of the aforementioned criteria, vitiligo was defined as “active”. VES (Vitiligo Extent Score, (van Geel et al., 2016) was also assessed for each patient; HOMA-IR (Homeostatic Model Assessment for Insulin Resistance) was calculated for each subject based on raw data. Patients with an already established diagnosis of diabetes, either type I OR II, were excluded from the present study. In total, 71 patients affected by vitiligo in the absence of a clinical diagnosis of diabetes were selected. Chi-Square Test, One-way ANOVA, Pearson and Spearman’s correlation test were used for statistical analysis. A p value < 0.05% was considered significant. No significant differences were detected in terms of sex distribution (M:36, F:35). Mean age was 32.8 (±18). Of note, approximately one-quarter of patients had pathological values HOMA-IR. Main patient characteristics are summarized in Table 1.

**TABLE 1:** Patient characteristics. Results are indicated as absolute frequency (N) or mean values and respective standard deviations (SD). Interestingly, relatively high variability was observed in VES scores (range: 0.080 - 3,716). NA: not available; VES: vitiligo extent score; HOMA-IR: Homeostatic Model Assessment for Insulin Resistance.

	N/mean (±SD)	%
Age	32.8 (±18.1)	-
Sex (M)	36	50.7%
Familiar history (+)	15 *(NA:8)*	23.8%
Active disease	34	47.9%
VES	0.702 (±0.078)	-
HOMA-IR > 2.5	13 *(NA:22)*	26.5%

Not surprisingly, VES score was found to directly correlate with patient age and disease duration (ρ = 0.292 and 0.425 respectively, p < 0.05%). Spearman’s correlation test also underscored higher VES scores to be positively associated with an altered HOMA-IR (p = 0.016).

**FIGURE 3:**
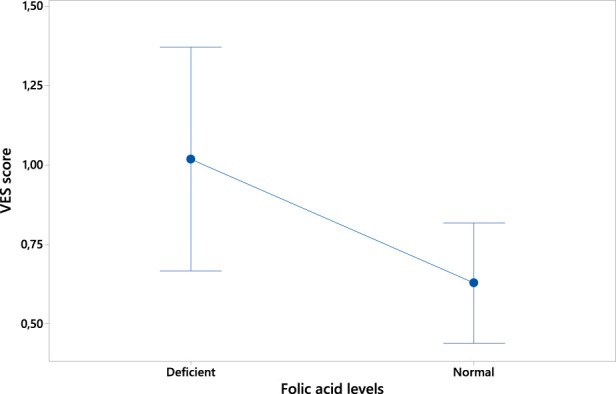
Plot of the inverse correlation between folic acid levels (expressed in ng/mL) and VES score.

On the contrary, folic acid levels were found to negatively correlate with VES score (ρ = 0.431 p = 0.006; see Figure 3).

In conclusion, our data strongly support the notion of metabolic impairment in patients affected by vitiligo, even in the absence of clinically evident diabetes. Moreover, alterations of folic acid levels in vitiligo patients confirm such alterations not to be limited to glucose metabolism.

## 6 Available treatments for vitiligo

There is currently no definitive cure for vitiligo. The most frequently used treatments have been for long topical corticosteroids, topical calcineurin inhibitors (TCIs), and phototherapy (Zubair and Hamzavi, 2020; Rosmarin et al., 2024). Laser is also considered a possible option; however, its application is limited because of its costs and its suitability limited to the treatment of small areas.

The global efficacy of these treatments is limited, even when used in combination, as also demonstrated by systematic reviews and meta-analyses (Lee J. H. et al., 2019; Kubelis-López et al., 2021; Feng J. et al., 2022; Feng et al., 2022 X.).

The recent advances in the understanding of vitiligo pathogenesis have promoted new therapeutic alternatives. Treatment of vitiligo with oral or topical Janus kinase inhibitors (JAKi), such as ruxolitinib and tofacitinib, seems to be particularly promising (Tavoletti et al., 2023; Utama et al., 2024). Recently, topical ruxolitinib has become available for the treatment of non-segmental vitiligo. JAKi interfere with the signal transducers and activators of the transcription (STAT) pathway, crucial for intracellular signaling of inflammatory mediators involved in immune function, including growth factors, interferons, and interleukins. The synergistic use of JAKi with phototherapy or sun exposure has also already been explored. Such combination treatment is supported by the hypothesis that repigmentation requires a dual approach, involving on the one hand immune suppression and, on the other hand, melanocyte stimulation.

Most of the available treatments for vitiligo, including JAKi, corticosteroids and TCI, rely on a common mechanism of action based on immune response inhibition. However, currently, no treatment aimed at improving the cellular metabolic status is currently available. Hypothetically, these strategies could reduce the production of ROS and, therefore, block the release of second messengers and auto-antigens, thus eliminating a crucial trigger for the activation of the immune system.

## 7 Therapeutic opportunities for vitiligo emerging from metabolic diseases

While current treatments focus on the immunological mechanisms involved in vitiligo pathogenesis, their efficacy remains suboptimal, prompting the search for new therapeutic options. Moreover, given the tendency to relapse after treatment discontinuation, current research focuses on preventing rather than counteracting immune system activation in vitiligo. The idea of targeting metabolic impairment (either clinical or subclinical) in vitiligo aims at preventing the accumulation of ROS and other inflammatory mediators, therefore blunting immune cell recruitment.

### 7.1 Targeting autophagic machinery

Given the emerging role of defective autophagic processes observed in vitiligo patients, promoting autophagy has emerged as a possible therapeutic approach. Autophagy is critical in degrading damaged cellular organelles and nutrient recycling. Nonetheless, dysregulation of autophagy may compromise cellular homeostasis and affect both, innate and adaptive immunity. Another important connection between autophagy and the innate immune response relies in cytokine release (Bhattacharya et al., 2013). Impaired autophagy determines IL-1β and IL-18 production by macrophages, thereby promoting the inflammasome activation (Saitoh et al., 2008). Dysregulated autophagy also disrupts the antioxidant defense system in vitiligo melanocytes (Qiao et al., 2016) making these cells more prone to senescence and cell death (Zhang et al., 2015; Qiao et al., 2020; Cui et al., 2022). By contrast, well-functioning autophagy prevents oxidative-stress induced cell death (Cui et al., 2022). Chronic up-modulation of autophagic markers in vitiligo melanocytes, keratinocytes and fibroblasts firmly demonstrated the shift to a persistent catabolic metabolism (Yang et al., 2022; Murase et al., 2013; Cui et al., 2022). In line with the notion that autophagy is a mechanism of metabolic surveillance, stable lesions exhibit enhanced autophagy compared to active vitiligo lesions, indicating such process to counteract the progression of the disease (Yu et al., 2021). Despite autophagy represents a survival strategy when reduced energy sources are available, it might also lead to senescence and autophagy-dependent cell death (ADCD) and autophagy-mediated cell death (AMCD) (Ploumi et al., 2022), possibly contributing to melanocytes disappearance.

The mammalian target of rapamycin (mTOR) is a suppressor of autophagy: rapamycin, an mTOR inhibitor, might therefore be a drug of interest. Of note, rapamycin treatment upregulated *in vitro* the expression of MITF and pro-melanogenic enzymes in melanocytes and melanoma cells (Nie et al., 2016; Hah et al., 2012). In mouse models of vitiligo, rapamycin has been shown to expand Tregs and inducing IL-10 production while decreasing IFN-γ and IL-6, therefore blocking the depigmentation process (Zhang et al., 2021). Topical rapamycin (0.1% or 0.001% cream) is currently under evaluation for its efficacy and safety in treating nonsegmental vitiligo lesions (NCT05342519).

### 7.2 Targeting lipid metabolism

A case report described a patient with vitiligo who re-pigmented under treatment with simvastatin, an inhibitor of HMG-CoA reductase, the rate-limiting enzyme in cholesterol synthesis, usually used to control blood lipid disorders (Noël et al., 2004). *In vitro*, simvastatin protects human melanocytes from H_2_O_2_-induced oxidative stress by activating Nrf2 (Chang et al., 2017). Simvastatin reversed depigmentation in mouse models due to inhibition of interferon-γ signalling by blocking the activation of STAT1/2 and lowering the number of infiltrating autoreactive CD8 T cells (Agarwal et al., 2015). However, two small clinical trials to test the use of oral simvastatin as a treatment for vitiligo failed to confirm its efficacy (Vanderweil et al., 2017; Zhang et al., 2021). Very recently, a clinical trial was designed to try out the topical application to achieve much higher concentrations of statins in the treated area without the risk of adverse effects associated with their systemic use. In this case, the authors concluded that a 1% simvastatin cream directly onto skin lesions, but not torvastatin, inhibits disease progression (Niezgoda et al., 2024). However, topical administration of both compounds did not allow for the achievement of significant re-pigmentation in vitiligo patients compared to vehicle treatments.

### 7.3 IGF-1

There is also a growing understanding of the importance of insulin and IGF-1 signaling in vitiligo. IGF-1 plays a pivotal role in the regulation of cell proliferation, differentiation and apoptosis. Insulin-like growth factors (IGFs) are important regulators of energy metabolism homeostasis and growth (Okuyama et al., 2021). Additionally, IGF-1 regulates mitochondrial mass and function and contributes to specific processes including ATP production (Poudel et al., 2020).

At high concentrations, IGF-1 activates the insulin receptor (Boucher, 2006). Thus, in states of insulin resistance, IGF-1 can regulate glucose metabolism. The amount of circulating insulin has been recently with the regulation of pigment patterns in zebrafish models, suggesting functional lineage-specific responsiveness to this hormone (Zhang et al., 2018). The biological action of IGFs is modulated by several IGF-binding proteins (IGFBPs) (Baxter, 2023). Our group previously demonstrated IGFBP3 and IGFBP7 downregulation in vitiligo melanocytes (Bellei et al., 2013). IGFBP3 and IGFBP7 have also been indicated as a hallmark of melanocytes SASPs, due to their capability of triggering senescence in melanoma cells (Wajapeyee et al., 2008; Naspi et al., 2014).

Hussein and collaborators recently reported lowered levels of IGF-1 in the skin and blood of vitiligo patients compared to reference samples, confirming the systemic nature of vitiligo (El-Komy et al., 2023). Collectively, data support further exploration concerning IGF-1 supplementation to treat vitiligo.

### 7.4 Mitochondria-targeted therapy for vitiligo

Existing data suggest that mitochondrial dysfunction plays a causative role in the development and progression of vitiligo. Mitochondrial dysfunction initiates intracellular signaling cascades that can impair many processes known to be important in the progression of the disease including autophagy, inflammation, and cellular responses to growth factors. ROS are byproducts of normal mitochondrial metabolism. Overproduction of ROS can occur due to limited scavenger capacity and because of abnormal intrinsic production. Both mechanisms have been documented in vitiligo (Chang and Ko, 2023) leading to H2O2 accumulation in the epidermis of patients. Therefore, targeting mitochondrial metabolism to treat vitiligo has also garnered great attention. Topical application of pseudocatalase has been proposed in the past as a potential agent to arrest disease progression (Schallreuter et al., 2008).

Elamipretide (MTP-131), a small mitochondrially-targeted tetrapeptide demonstrated beneficial effects in maintaining cellular biogenetics and preventing reactive oxygen species-induced cell damage by the cardiolipin-cytochrome c complex. MTP-131 inhibits lipid peroxidation and prevents mitochondrial swelling, aiding mitochondrial protection (Miyamoto et al., 2020) and is currently being explored for its potential to treat several morbid conditions (neuropathy, diabetic kidney disease, myocardial injury, Alzheimer disease and impaired wound healing) (Miyamoto et al., 2020; Tse et al., 2020; Swain et al., 2024). Despite still being under investigation, MT-131 represents a novel and potentially transformative therapy for vitiligo, addressing both the autoimmune and oxidative components of the disease. If successful, it could fill an unmet need in vitiligo management.

### 7.5 PPARγ agonists as potential therapeutic tools for vitiligo

PPARs are fatty acid-activated transcription factors, part of a nuclear hormone receptor superfamily, that regulate energy metabolism (Christofides et al., 2021). Three PPAR subtypes have been identified so far: PPARα, PPARγ, and PPARβ/δ. In addition to tissues regulating whole-body energy homeostasis, PPARs are expressed in immune cells and have an emerging critical role in immune cell differentiation and fate commitment. Specifically, PPAR-γ has a well-established role in the regulation of gene expression for processes such as glucose homeostasis, cellular differentiation and apoptosis (Qi et al., 2000). In the skin, PPARγ is essential for maintaining skin barrier permeability, regulating keratinocyte proliferation/differentiation, and modulating antioxidant responses upon ligand binding. Therefore, PPARγ activation has important implications for skin homeostasis. PPARα and PPARδ mostly regulate genes involved in substrate delivery, oxidative phosphorylation, and energy homeostasis. In contrast, PPARγ is more important in lipogenesis and lipid synthesis and is highly expressed in the white adipose tissue. PPARγ is considered one of the master modulators of mitochondrial biogenesis and function and plays a crucial role in fat cell differentiation, lipid storage, vascular function, and energy metabolism (Ahmadian et al., 2013).

Thiazolidinediones (TZDs) represent a class of compounds that are high-affinity ligands for the transcription factor PPARγ (Villacorta et al., 2009). Among the TZD, Pioglitazone (PGZ) is a 10-fold less potent PPARγ agonist compared to other commercially available TZD and is as effective as an insulin sensitizer, but with a greater safety margin. Thus, PGZ is frequently used to improve glycemic control and reduce insulin resistance in patients affected by type 2 diabetes. Although Pioglitazone was initially thought to act only as a direct transcriptional regulation of the PPARγ nuclear receptor, later data revealed that it also binds to mitochondrial membranes (Colca et al., 2004).

Studies conducted in multiple cell types demonstrated that TZDs inhibit mitochondrial pyruvate carrier (MPC) activity by direct binding (Divakaruni et al., 2013; McCommis and Finck, 2015). Reduced mitochondrial entry of pyruvate through the MPC results in enhanced use of other energetic substrates, including fatty acids and amino acids (McCommis and Finck, 2015; Divakaruni et al., 2013; Buchanan and Taylor, 2020), which may mediate some of the beneficial effects of PGZ a variety of diseases including vitiligo. MPC inhibition modulates the activity of various energy-sensing signaling cascades such as AMP-activated protein kinase (AMPK), the mammalian target of rapamycin (mTOR) kinase with a direct implication in most pathogenic mechanisms of vitiligo including inflammation, and autophagy.

PGZ has also been demonstrated to decrease the production of inflammatory mediators, such as Interleukin 6 (IL-6), therefore acting as an immune-modulating agent (El-Gharabawy et al., 2017). For this reason, the therapeutic potential of this drug has already been investigated in other chronic immune-mediated conditions, such as psoriasis (Bongartz et al., 2005; Lin et al., 2022).

A potential role for PGZ in the scenario of vitiligo has been recently postulated based on the observation of its impact on glucose metabolism in melanocytes and fibroblasts from vitiligo patients. Bioinformatic analyses also confirmed PPARγ modulators as possible innovative approaches for vitiligo.

PGZ improves cellular energetic status by increasing mitochondrial membrane potential and ATP production while reducing ROS levels. More in detail, PGZ induced the expression of anaerobic glycolytic enzymes and due to the improvement of ATP production reduced the gene expression of glucose transporters Glut1 and Glut4. Concomitantly, *in vitro*, treatment of vitiligo melanocytes with PGZ reduced the expression of enzymes involved in β-oxidation and the Krebs cycle, while up-regulating antioxidant enzymes. In parallel, PGZ reduced the expression of voltage-dependent anion-selective channel 1 (VDAC1) uncoupling protein 2 (UCP2), which suggests improved mitochondrial efficiency. Finally, PGZ was shown to reduce the expression of pro-inflammatory mediators, SASPs and PD-L1, suggesting a potential role in reducing inflammation and immune dysregulation in vitiligo. Overall, PGZ shows promise in modulating glucose metabolism, improving mitochondrial function, and reducing the inflammatory milieu.

### 7.6 Other drugs potentially useful in vitiligo

Metformin, a widely used anti-diabetic drug, is gaining attention for its role in treating immune-mediated disorders due to its immunomodulatory and anti-inflammatory properties (Schuiveling et al., 2018). Insulin resistance has been linked to an increased risk of vitiligo and psoriasis, suggesting that metformin may have systemic benefits in chronic immune-mediated diseases (Sung et al., 2020; Huang et al., 2023). Given its favourable side-effect profile and affordability, metformin should be used as an adjunctive therapy for vitiligo and other skin conditions like psoriasis or atopic dermatitis, in case co-existing metabolic syndrome or insulin resistance (Badr et al., 2013; Gouveri and Papanas, 2023). Thus, very recently, a phase II clinical trial proposing daily orally administrated metformin has been approved by FDA (NCT05607316). Beyond its traditional use in managing type 2 diabetes by improving insulin sensitivity, in fact, metformin holds the potential to modulate immune responses through the activation of AMP-activated protein kinase (AMPK) and inhibition of the mTOR pathway (Chen et al., 2022). These pathways are crucial in regulating immune cell metabolism, proliferation, and function. In autoimmune and inflammatory conditions such as systemic SLE, psoriasis, rheumatoid arthritis, and multiple sclerosis, metformin has been shown to reduce disease activity by decreasing pro-inflammatory cytokine production and altering T-cell metabolism (Sung et al., 2020; Gouveri and Papanas, 2023). By shifting T cells towards a regulatory phenotype, metformin helps attenuating the pathogenic immune responses that drive chronic immune-mediated skin conditions.

Fibrates are a class of drugs primarily used to manage dyslipidemia that activate the PPAR-α (Bougarne et al., 2018). In vitiligo, oxidative stress and immune-mediated destruction of melanocytes are key pathogenic mechanisms: PPAR-α activation can reduce oxidative stress by enhancing the activity of antioxidant enzymes such as superoxide dismutase and catalase, therefore protecting melanocytes from oxidative damage (Abulaban et al., 2024). Additionally, fibrates have been shown to modulate immune responses by decreasing pro-inflammatory cytokines like TNF-α and IFN-γ, which are implicated in vitiligo pathogenesis (Zhang et al., 2022).

Given their well-established use in cardiovascular medicine and their favorable safety profile, fibrates could emerge as a novel adjunctive therapy for vitiligo, especially in patients with coexisting lipid abnormalities or metabolic syndrome.

## 8 Discussion

The present work sheds light on the possible metabolic factors playing a role in vitiligo pathogenesis, both at a cellular and a systemic level. Main findings are summarized in Figure 3.

Current literature suggests a strict relationship between immune-mediated disorders and metabolic syndrome. Vitiligo is epidemiologically connected with diabetes type I and type II suggesting that both autoimmune and non-autoimmune components are involved in the pathogenesis (Lyu and Sun, 2022). Moreover, several systemic markers associated with metabolic abnormalities like glucose intolerance and lipid abnormalities have already been widely demonstrated in subjects with vitiligo.

Despite different strategies are currently available, the management of vitiligo remains challenging. The switch from an immunologically centered pathogenetic hypothesis to a metabolic-based theory would represent a groundbreaking change in the setting of vitiligo for its therapeutic implications.

Most of the available treatments for vitiligo rely on the inhibition of the immune response as a curative mechanism, with an IFNy-dependent CD8^+^ cytotoxic response being postulated to play a major role in melanocyte damage. However, immunosuppressant treatments, either topical or systemic, are frequently accompanied by a series of non-negligible side effects (e.g., skin thinning, increased rate of infections). Thus, for a chronic disease such as vitiligo, there is an urgent need for new preventive strategies. Targeting glucose and/or lipid metabolism with already available molecules could prevent metabolism-associated danger signals and auto-antigen release. In particular, the repurposing of PPARy agonists in the setting of vitiligo is supported by preclinical *in vitro* studies, as well as by bioinformatic tools. Moreover, the improvement of the metabolic balance and the retrenchment of oxidative stress can provide health benefits beyond the skin, such as better glucose metabolism and overall wellbeing, even in the absence of immediate improvement in skin pigmentation.

While oral antidiabetics and lipid-lowering agents offer potential therapeutic benefits for vitiligo by modulating oxidative stress and inflammation, their use presents limitations (Feng J. et al., 2022). Metformin may enhance redox balance but carries risks of gastrointestinal distress, lactic acidosis and vitamin B12 deficiency (Bouchoucha et al., 2011; Akhter and Uppal, 2020). Thiazolidinediones influence immune pathways yet are associated with weight gain and cardiovascular risks (Schernthaner and Chilton, 2010). Statins, despite their anti-inflammatory properties, may induce myopathy, hepatic dysfunction, and, in rare cases, skin eruptions (Vesza et al., 2018; Lagunas-Rangel et al., 2024).

In conclusion, drug repurposing appears to be an interesting option for vitiligo patients, especially with regards to therapies already approved and prescribed for metabolic disturbances. However, further studies are needed for a thorough understanding of metabolic agents’ risks and benefits in this setting.

## Data Availability

The raw data supporting the conclusions of this article will be made available by the authors, without undue reservation.
